# Improvements in Plasma Tumor Necrosis Factor-Alpha Levels after a Weight-Loss Lifestyle Intervention in Patients with Obstructive Sleep Apnea

**DOI:** 10.3390/life12081252

**Published:** 2022-08-17

**Authors:** Michael Georgoulis, Nikos Yiannakouris, Roxane Tenta, Ioanna Kechribari, Kallirroi Lamprou, Emmanouil Vagiakis, Meropi D. Kontogianni

**Affiliations:** 1 Department of Nutrition and Dietetics, School of Health Sciences and Education, Harokopio University of Athens, 70 Eleftheriou Venizelou Str., 17676 Athens, Greece; 2 Center of Sleep Disorders, 1st Department of Critical Care, Evangelismos General Hospital, 45-47 Ipsilantou Str., 10676 Athens, Greece

**Keywords:** sleep-disordered breathing, inflammation, tumor necrosis factor alpha, obstructive sleep apnea, weight loss, dietary intervention, lifestyle intervention, Mediterranean diet

## Abstract

Obstructive sleep apnea (OSA) and systemic inflammation typically coexist within a vicious cycle. This study aimed at exploring the effectiveness of a weight-loss lifestyle intervention in reducing plasma tumor necrosis factor-alpha (TNF-a), a well-established modulator of systematic inflammation in OSA. Eighty-four adult, overweight patients with a diagnosis of moderate-to-severe OSA were randomized to a standard care (SCG, *n* = 42) or a Mediterranean lifestyle group (MLG, *n* = 42). Both groups were prescribed continuous positive airway pressure (CPAP), while the MLG additionally participated in a 6-month behavioral intervention aiming at healthier weight and lifestyle habits according to the Mediterranean pattern. Plasma TNF-a was measured by an immunoenzymatic method both pre- and post-intervention. Drop-out rates were 33% (*n* = 14) for the SCG and 24% (*n* = 10) for the MLG. Intention-to-treat analysis (*n* = 84) revealed a significant decrease in median TNF-a only in the MLG (from 2.92 to 2.00 pg/mL, *p* = 0.001). Compared to the SCG, the MLG exhibited lower follow-up TNF-a levels (mean difference adjusted for age, sex, baseline TNF-a and CPAP use: −0.97 pg/mL, *p* = 0.014), and further controlling for weight loss did not attenuate this difference (*p* = 0.020). Per protocol analyses (*n* = 60) revealed similar results. In conclusion, a healthy lifestyle intervention can lower plasma TNF-a levels in patients with OSA.

## 1. Introduction

Obstructive sleep apnea (OSA) and systemic inflammation typically coexist and combinedly contribute to increased cardiovascular risk [1]. Continuous positive airway pressure (CPAP) is currently the first line treatment for OSA, but its effectiveness in improving patients’ inflammatory profile remains questionable [1]. Although weight loss and the adoption of a healthy lifestyle may reduce inflammation [2], only a few interventional studies have explored the anti-inflammatory benefits of lifestyle modification in patients with OSA, showing improvements in C-reactive protein (CRP) levels [3,4]. Besides CRP, tumor necrosis factor-alpha (TNF-a) is a well-established modulator of systematic inflammation and has been proposed as a key biomarker for the onset and progression of OSA; chronic intermittent hypoxia, a key characteristic of respiratory pathology in OSA, can induce TNF-a expression and lead to cardiovascular complications, while TNF-a inhibition has been shown to ameliorate OSA progression [5].

In previous reports of the MIMOSA (Mediterranean diet/lifestyle Intervention for the Management of Obstructive Sleep Apnea) randomized controlled clinical trial [6,7,8], we demonstrated that the combination of a 6-month weight-loss dietary/lifestyle intervention based on the Mediterranean pattern with the prescription of CPAP therapy can lead to greater improvements in OSA patients’ polysomnographic indices, symptoms and cardiometabolic manifestations, including reductions in high-sensitivity CRP, compared to CPAP alone; we additionally showed that a Mediterranean lifestyle intervention, a holistic approach for beneficial dietary, physical activity and sleep modification according to the Mediterranean pattern, is superior in improving several sleep-related, cardiometabolic and oxidative stress indices in patient with OSA compared to the Mediterranean diet per se. In the present study we aimed at further exploring the potential benefits of this weight-loss Mediterranean lifestyle intervention in ameliorating systemic inflammation in OSA through improvements in plasma TNF-a levels.

## 2. Materials and Methods

This manuscript presents secondary analyses of the MIMOSA trial (registered at ClinicalTrials.gov, identifier: NCT02515357) in a subsample with available plasma TNF-a measurements. The study protocol has been previously presented in detail [6,7,8] and was approved by the Ethics Committee of Harokopio University. The study population consisted of 84 newly-diagnosed, adult, overweight male and female patients with a polysomnography (PSG)-extracted apnea-hypopnea index (AHI) ≥ 15 events/h (indicative of moderate-to-severe OSA), who provided a signed written consent and were randomly allocated to a standard care group (SCG, *n* = 42) or a Mediterranean lifestyle group (MLG, *n* = 42). Patients of both groups were prescribed CPAP therapy as the standard care for OSA management. Specifically, following the initial diagnostic PSG, participants were subjected to an overnight in-laboratory CPAP titration sleep study and were accordingly prescribed the same auto-CPAP device on the basis of current recommendations for OSA management [9,10]. Patients were asked to obtain the prescribed auto-CPAP device, were given detailed information on CPAP technical standards, cleaning and maintenance and were instructed to use it daily during night sleep for the whole study period.

On top of CPAP, patients in the MLG also participated in a 6-month lifestyle modification program aiming at a healthier body weight, improving dietary habits towards a Mediterranean-style diet (i.e., a dietary pattern characterized by abundance of fruits, vegetables, non-refined grains, legumes, nuts and seeds; olive oil as the principal fat source; moderate consumption of dairy products, white meat and fish/seafood; prudent alcohol intake; limited amounts of red meat and the avoidance of processed foods, such as sweets, sugar-sweetened beverages and fast food) [11], the adoption of a physically active lifestyle (≥150 min/week of any kind of lifestyle physical activity or organized exercise) [12] and optimal night-time sleep duration (7–9 h/day) [13]. The lifestyle intervention was structured in seven, 60-min, group (3–5 patients) counselling sessions led by the research dietitian, performed biweekly for the first two months and then monthly for the next four months of the study. In brief, the first session was devoted to weight loss and emphasis was given to dietary practices that can help reduce energy intake, such as food portion control, correct identification of hunger and satiety and proper meal conditions. Patients were also provided with pedometers and were asked to record their total daily steps, aiming at a gradual increase with the ultimate goal of 10,000 steps/day. In the following six sessions, patients were gradually trained to increase adherence to the principles of the Mediterranean lifestyle. In each session patients were informed about the nutritional value and health effects of specific food groups and were given goals about their optimal consumption according to the Mediterranean diet pyramid [11]. Other healthy dietary/lifestyle practices, such as ensuring a nutritional variety, choosing unprocessed, traditional, local and seasonal Mediterranean foods; implementing healthy cooking techniques; adopting a physically active lifestyle with emphasis on outdoor convivial activities; and sleep hygiene were also addressed. The intervention was based on cognitive-behavioral therapy, with emphasis on goal setting, problem solving, self-monitoring of lifestyle habits (patients were asked to record specific lifestyle parameters in self-monitoring print forms on a daily basis throughout the 6-month intervention, such as the consumption of major food groups, the duration of physical activity and the duration of night-sleep, in order to evaluate adherence to intervention goals and enhance motivation), stimulus control, managing high-risk situations and relapse prevention to facilitate behavioral change [14,15]. At the beginning of each session, the research dietitian weighed the patients, reviewed their self-monitoring forms and patients were asked to report difficulties/barriers in achieving the lifestyle goals set at the previous session. Then, patients were led in a group discussion to create a plan for dealing with the difficulties/barriers described. In the second part of each session, new lifestyle goals were set, the adherence to which was evaluated in the next session.

Participants’ anthropometric indices, lifestyle habits and 12-h fasting plasma TNF-a levels were assessed pre- and post-intervention. Body weight (kg) and height (m) were measured following a standardized protocol; the body mass index (BMI) was calculated as weight divided by height squared, and participants were classified as overweight or obese according to international BMI cut-off points. Dietary habits, in terms of habitual food/food group consumption were evaluated through a food frequency questionnaire previously validated in the adult Greek population [16], and adherence to the Mediterranean diet was evaluated through the Mediterranean Diet Score (MedDietScore) [17]. The MedDietScore is an a priori index, taking into account the habitual consumption of nine food groups (i.e., whole grains, potatoes, fruits, vegetables, legumes, full-fat dairy products, fish, poultry, and red meat and products), olive oil and alcohol. Based on the recommendations of the Mediterranean diet, the consumption of each of the 11 components of the index is scored using a scale that ranges from 0 to 5. For foods typical of the Mediterranean diet, i.e., whole grains, potatoes, fruits, vegetables, legumes, fish and olive oil, scoring ranges from 0 to 5 for a very rare to a very frequent consumption, respectively, while the opposite scale (i.e., 0 for a very frequent to 5 for a very rare consumption) is used for foods not typically consumed in the Mediterranean diet, i.e., full-fat dairy products, poultry and red meat. Alcohol consumption was given a score of 0 for no consumption or consumption of >7 standardized servings per day, and scores of 1 to 5 for the consumption of 6–7, 5–6, 4–5, 3–4 and <3 standardized servings per day, respectively (1 standardized serving equals to 12 g of ethanol). The total MedDietScore ranges from 0 to 55, with higher values indicating a greater level of adherence to the Mediterranean diet. The short-form of the International Physical Activity Questionnaire [18] was used to evaluate physical activity habits, and total daily time (min/day) of physical activity was calculated for each participant. Daily duration (h/day) of night sleep and CPAP use were self-reported by participants. TNF-a levels were measured by an immunoenzymatic method (Human TNF-alpha Quantikine ELISA Kit, R&D Systems, Minneapolis, MN, USA); the intra-assay and inter-assay variation coefficients were <5% and <8%, respectively.

The MIMOSA trial was originally powered to detect a significant effect size of the lifestyle intervention on the AHI in a population of 180 patients. For the purpose of the present secondary analysis, post hoc power calculation revealed that the study subsample (*n* = 84) was sufficient to obtain ≥80% power to detect a difference in follow-up TNF-a levels between the SCG and the MLG, allowing for a type-I error rate of 0.05. The intention-to-treat method [19] was applied in the primary analyses and a secondary per protocol analysis was also performed. Analyses were conducted using the SPSS software version 23 (IBM Corp. 2015, Armonk, NY, USA) and *p*-values < 0.050 indicated statistically significant results. The Shapiro–Wilk test was used to assess the normality of continuous variables. Differences between groups were tested through the chi-square test for categorical variables or the Student’s t-test and the Mann–Whitney U test for normal and skewed continuous variables, respectively. Changes from baseline within each group and differences between groups in plasma TNF-a were tested through the Wilcoxon signed-rank test and the Mann–Whitney U test, respectively. The analysis of covariance was applied to explore mean differences (MD) and 95% confidence intervals (CI) between groups in TNF-a at the 6-month follow-up; age, sex, baseline values of TNF-a, CPAP use (h/day), %Δ weight [((follow-up weight − baseline weight)/baseline weight) × 100] and follow-up AHI levels served as covariates.

## 3. Results

The trial flow diagram of the MIMOSA study can be found in previously published reports [6,7,8]. For the needs of the present secondary analyses, 84 newly-diagnosed patients with moderate-to-severe OSA originally randomized in two groups (*n* = 42 in the SCG and *n* = 42 in the MLG) and for whom plasma TNF-a measurements were available, consisted the final study population. Of those, 24 patients were lost to follow-up, 14 (33%) in the SCG and 10 (24%) in the MLG. The main reasons for study discontinuation in the MLG were circumstances or events that made participation in the counselling sessions unfeasible, the lack of interest in the intervention, group-session scheduling conflicts or the unjustified complete loss of contact with the research dietitian. Participants in the SCG were considered dropouts if they did not complete the 6-month re-evaluation (e.g., could not be reached to schedule an appointment, refused to participate in the follow-up due to lack of time or interest or did not show up for their scheduled appointment).

The baseline characteristics of the study population are shown in Table 1. Mean age was 47 ± 9 years, males accounted for 81% of the study sample and obesity prevalence was 82%. Participants exhibited a moderate adherence to the Mediterranean diet (mean MedDietScore: 31.9 ± 4.6), low physical activity level [median (1st, 3rd quartile) min/day: 12.9 (4.29, 34.3)] and an inadequate mean daily night-time sleep duration (6.1 ± 1.5 h/day, compared to the recommended 7–9 h/day for adults). The median (1st, 3rd quartile) AHI value was 58.0 (26.0, 89.0) events/h and 74% of participants had an AHI ≥ 30 events/h, indicative of severe disease. Although all enrolled patients were prescribed with CPAP, 86% of the SCG and 79% of the MLG acquired the device and started the treatment at baseline (*p* = 0.393). Among users, mean daily CPAP use was 4.32 ± 2.45 h/day in the SCG and 3.41 ± 2.45 h/day in the MLG (*p* = 0.128). No significant differences between the SCG and the MLG were observed in baseline sociodemographic, lifestyle and clinical characteristics.

At the 6-month follow-up, mean percent body weight change was 0.16 ± 2.91 % in the SCG and −11.1 ± 5.90 % in the MLG (*p* < 0.001). Patients in the SCG did not present any significant changes in lifestyle habits (data not shown); on the contrary, mean MedDietScore (from 31.7 ± 4.7 to 40.9 ± 3.9), median (1st, 3rd quartile) daily physical activity time [from 16.0 (5.71, 34.5) to 50.8 (42.9, 63.6) min/day] and mean sleep duration (from 6.3 ± 1.6 to 7.1 ± 0.57 h/day) increased in the MLG (all *p* ≤ 0.001), with a significant difference compared to the SCG (all *p* < 0.001). According to the intention-to-treat analysis (*n* = 84), a decrease in median TNF-a levels was observed in the MLG (*p* = 0.001) but not in the SCG (*p* = 0.975) (Table 2). Median absolute and percent reduction of TNF-a was also higher in the MLG compared to the SCG (both *p* < 0.050). Post-intervention age-, sex-, baseline- and CPAP use-adjusted levels of TNF-a were lower in the MLG compared to the SCG [MD (95%CI): −0.97 (−1.74, −0.20) pg/mL, *p* = 0.014] and the difference between groups was not attenuated after further adjustment for %Δ weight (*p* = 0.020). When 6-month AHI was also included as a covariate in the model, the difference in TNF-a remained marginally significant (*p* = 0.050). Per protocol analyses (*n* = 60) revealed similar results (Table 2).

## 4. Discussion

In the present study, the effect of a weight-loss lifestyle intervention on plasma TNF-a levels was explored in an adult, overweight population of patients with OSA of at least moderate-severity. Although CPAP alone did not have a significant effect on TNF-a, a meaningful reduction of approximately 1 pg/mL was achieved when CPAP was combined with a feasible behavioral intervention aiming at a healthier body weight through the adoption of the Mediterranean lifestyle. This is in line with previous clinical trials showing improvements in TNF-a after healthy lifestyle interventions in individuals of increased cardiometabolic risk (e.g., those with metabolic syndrome) and patients with diabetes mellitus type 2 and cardiovascular disease, conditions which are tightly linked to OSA from a pathophysiological point of view [20,21]. Interestingly, the observed reduction in TNF-a was independent of weight loss and can be partly attributed to the strong anti-inflammatory properties of a healthy lifestyle, combining a Mediterranean-style diet [22] with daily physical activity [23] and adequate sleep duration [24].

OSA is tightly and bidirectionally linked to systemic inflammation [1]. Although several inflammatory markers have been positively associated with the presence of OSA, TNF-a in particular has emerged as a clinically-useful index for predicting OSA risk, with patients exhibiting higher TNF-a levels compared to healthy controls, and TNF-a values increasing as OSA severity progresses from mild to severe [5]. Given that TNF-a is implicated in cardiovascular pathophysiology [25], interventions for normalizing TNF-a values are important in OSA, which is currently recognized as a disease of cardiometabolic nature [26]. In this context, although CPAP remains the gold-standard therapy for OSA, its effectiveness in ameliorating inflammation remains controversial; some previous clinical trials have revealed that CPAP therapy can lead to significant reductions in markers of inflammation (e.g., CRP, TNF-a and interleukin-6) in patients with OSA [27], whereas other studies, similarly to our findings, have not reported a significant anti-inflammatory effect [28]. The observed lack of a significant effect of CPAP on TNF-a is in line with theories proposing that OSA represents a manifestation of the metabolic syndrome [29,30]. In this context, it can be speculated that chronic subclinical inflammation, a typical component of the metabolic syndrome, pre-exists and contributes to the onset and progression of OSA, combined with central obesity, insulin resistance and oxidative stress. Although the cause–effect link between inflammation and OSA requires further investigation, it is possible that the inflammatory state in OSA is not merely a consequence of sleep-disordered breathing and intermittent hypoxia, and therefore cannot be entirely reversed through CPAP, which aims at normalizing breathing during sleep. This is also supported by the fact that the difference between groups in TNF-a observed in our study was partly attenuated but remained marginally significant after adjustment for residual AHI; this observation suggests that improvements in OSA severity can only partially explain improvements in inflammation and that healthy lifestyle interventions can both improve OSA severity and ameliorate inflammation through other mechanisms, including beneficial effects on body weight status and the pathophysiology of the metabolic syndrome [31]. The suboptimal use of CPAP by study participants, which is in line with previously published data [32], is another possible explanation for the lack of a significant change in TNF-a levels in the SCG.

To our best knowledge, this is the first interventional study to explore the effects of a weight-loss lifestyle intervention on plasma TNF-a levels in OSA. The study design (randomized controlled clinical trial); the diagnosis of OSA through an attended overnight in-hospital PSG; the evaluation of patients’ inflammatory status through TNF-a, which is considered a key modulator of systemic inflammation and an important biomarker for the onset and progression of OSA; and the implementation of a well-designed, feasible, multicomponent behavioral lifestyle intervention based on the health-promoting Mediterranean pattern are strong points of the present work. Limitations of our study include: the fact that this was a secondary analysis in a subsample of the original study population with available TNF-a measurements; the small sample size (*n* = 84) and the relatively high drop-out rate (*n* = 24, 28.5% of the study population), although we partially compensated for attrition by implementing intention-to-treat analysis and achieved sufficient statistical power for analyses; the fact that CPAP was prescribed and not provided to patients, which could explain its suboptimal use (82% of the study population, mean use: 3.89 ± 2.47 h/day), although the parentage of CPAP users was similar between study groups and the daily duration of CPAP use was included as a covariate in analyses; and the fact that our study was implemented in Greece, a typical Mediterranean country, in which the Mediterranean diet could be more easy to adopt, well-accepted by patients and sustainable and therefore our results cannot be universally generalized in the whole OSA population, especially patients in non-Mediterranean countries.

In conclusion, our findings support that healthy lifestyle interventions can be an efficient approach for improving OSA-related systemic inflammation and should be further tested and confirmed in future clinical trials incorporating adequate samples of OSA patients of different ethnic, sociodemographic and lifestyle backgrounds.

## Figures and Tables

**Table 1:** Baseline characteristics of the study population.

	Total (*n* = 84)	SCG (*n* = 42)	MLG (*n* = 42)	*p* ^a^
Age, years	46.5 ± 9.4	46.5 ± 9.4	46.5 ± 9.5	0.991
Male sex, *n* (%)	68 (81)	35 (83)	33 (79)	0.578
BMI, kg/m^2^	35.5 ± 5.5	35.6 ± 5.4	35.5 ± 5.7	0.953
Obesity, *n* (%) ^b^	69 (82)	34 (81)	35 (83)	0.776
MedDietScore (0–55) ^c^	31.9 ± 4.6	32.1 ± 4.6	31.7 ± 4.7	0.709
Physical activity, min/day	12.9 (4.29, 34.3)	10.7 (0.00, 34.3)	16.0 (5.71, 34.5)	0.296
Sleep duration, h/day	6.1 ± 1.5	5.9 ± 1.4	6.3 ± 1.6	0.183
AHI, events/h	58.0 (26.0, 89.0)	52.0 (28.5, 87.0)	63.5 (21.0, 94.0)	0.989
Severe OSA, *n* (%) ^d^	62 (74)	32 (76)	30 (71)	0.666
CPAP therapy, *n* (%)	69 (82)	36 (86)	33 (79)	0.393

Normally distributed and skewed continuous variables are presented as mean ± standard deviation and median (1st, 3rd quartile), respectively, while categorical variables are presented as absolute number (relative frequency). ^a^ Based on the chi-square test for categorical variables or the Student’s *t*-test and the Mann–Whitney U test for normal and skewed continuous variables, respectively. *p*-values < 0.050 indicate statistically significant differences. ^b^ BMI ≥ 30 kg/m^2^. ^c^ The score ranges from 0 to 55; higher values indicate higher adherence to the Mediterranean diet. ^d^ AHI ≥ 30 events/h of sleep. AHI, apnea-hypopnea index; BMI, body mass index; CPAP, continuous positive airway pressure; MedDietScore, Mediterranean diet score; MLG, Mediterranean lifestyle group; OSA, obstructive sleep apnea; SCG, standard care group.

**Table 2:** Changes within groups and differences between groups in plasma TNF-a levels.

Intention-to-Treat Analysis (*n* = 84)					
	SCG (*n* = 42)	*p* ^a^	MLG (*n* = 42)	*p* ^a^	*p* ^b^
TNF-a BL (pg/mL)	2.98 (1.42, 4.09)	0.975	2.92 (2.37, 4.00)	0.001	0.986
TNF-a FU (pg/mL)	2.90 (2.58, 3.11)		2.00 (0.92, 3.30)		0.009
Δ TNF-a (pg/mL)	0.13 (−1.58, 1.78)		−0.73 (−2.24, 0.14)		0.029
%Δ TNF-a	11.1 (−36.3, 108)		−25.0 (−67.1, 6.85)		0.004
**Per Protocol Analysis (*n* = 60)**					
	**SCG (*n* = 28)**	***p* ^a^**	**MLG (*n* = 32)**	***p* ^a^**	***p* ^b^**
TNF-a BL (pg/mL)	2.96 (1.30, 3.90)	0.685	2.97 (2.42, 4.18)	0.006	0.575
TNF-a FU (pg/mL)	2.90 (1.62, 3.60)		2.27 (0.79, 3.38)		0.283
Δ TNF-a (pg/mL)	0.63 (−1.29, 1.79)		−1.02 (−2.30, 0.28)		0.049
%Δ TNF-a	29.7 (−33.0, 117)		−29.5 (−70.0, 4.68)		0.015

Data are presented as median (1st, 3rd quartile). ^a^ Based on the Wilcoxon signed-rank test. *p*-values < 0.050 indicate statistically significant changes. ^b^ Based on the Mann–Whitney U test. *p*-values < 0.050 indicate statistically significant differences. BL, baseline; FU, follow-up; MLG, Mediterranean lifestyle group; SCG, standard care group; TNF-a, tumor necrosis factor-a; (%)Δ, (percent) change.

## Data Availability

Deidentified participant data and the study protocol will be made available by the corresponding author upon request.
